# Service product family optimization design for demand-driven older adult home care

**DOI:** 10.3389/fpubh.2024.1479586

**Published:** 2024-11-20

**Authors:** Chao Yu, Pengfei Zhao

**Affiliations:** School of Management, Shenyang University of Technology, Shenyang, China

**Keywords:** older adult home care services, product family design, optimization, genetic algorithm, Kano model

## Abstract

**Introduction:**

Diversification of demand leads to a wide range of older adults home care services and services market flexibility. In order to quickly respond to changing market demand, during the standard functioning of individual services, enterprises will choose the optimal combination of packages to meet the market’s expected demand and preferences to ensure that the enterprise can be in the uncertainty of the market regulation and has a relatively stable source of customers. The ability of companies to design older adults care service product families based on their operational strategy and financial position, thereby increasing their ability to enter emerging markets, is a key challenge.

**Methods:**

Firstly, the demand of the initial older adults group is obtained. Secondly, the attributes of each service item are classified using the Kano model. Finally, the actual operation constraints of the enterprise are incorporated, taking into account customer preference. Critical configuration problems such as module type, customer demand, probability of selection, and cost are also considered. The genetic algorithm is used to maximize the enterprise profit while considering customer preference. The efficacy of the approach is illustrated by the design exemplification of an older adult home care services firm.

**Results:**

This research suggests a novel method to product family design for older adults care services. The method determine the optimal older adults home care service product architecture system.

**Discussion:**

The article discusses three aspects of model parameters, potential constraints and extended application of the model for different contexts to expand the applicability and relevance of the study.

## 1 Introduction

The proportion of individuals aged 65 and above in China’s total population is steadily rising. As of 2023, this age group accounts for 15.4% of the population. With the aging trend accelerating, it is projected that by approximately 2031, the proportion of individuals aged 65 and above will exceed 20%, marking the transition into a super-aging society (1). The issue of aging has emerged as a significant concern in the rejuvenation of the Chinese people. Focusing on this matter will undoubtedly facilitate comprehensive economic and social transformation. Home care service is a type of assistance society offers to older individuals who live at home to address the challenges they face in their everyday lives. Based on survey data from the China Health and Wellness Commission, China’s older adults care pattern can be summarized as “9,073.” This means that around 90% of the older adult population live in their own homes, 7% depend on community-based care, and 3% reside in older adults care institutions. Aging in place is the predominant form of aging in China. Consequently, there is a substantial need for home care services for older people at home (2, 3). In China, home care facilities’ operational modes are primarily categorized into four types: government-funded operation, privately funded operation, government-funded private operation, and civilian-operated public assistance (4). Government-funded operation denotes an establishment funded, managed and administered by the government. Civilian-operated public assistance entails the government’s selection of appropriate service providers via open tender, wherein the government subsidizes but does not engage in the operational management of these providers. These two models are non-profit and primarily focused on low-income older individuals in poor physical condition with limited flexibility, predominantly providing low-cost or complimentary aged care services, lacking universality. The government-funded private operation model denotes a government-funded establishment operated by the private sector for profit. However, it struggles to deliver the leisure-oriented aged care services sought by seniors, resulting in incomplete coverage. The model is appropriate for the initial phase of aging development, characterized by market-oriented self-financing. However, as the government supplies hardware facilities, investors may refrain from augmenting their investment during operations, potentially compromising service quality. The privately funded operation model denotes the private sector financing a venture’s initiation and operation. The model offers substantial growth potential, is capable of addressing the comprehensive needs of the older adult population, exhibits high flexibility, and guarantees that the service retains the structural integrity of the product while complying with national requirements to accommodate the increasing demands of aging individuals. Consequently, the privately established and managed home care model possesses more significant potential for advancement.

In actuality, the majority of privately funded operation older adults care institutions function independently (2, 4). This study seeks to delineate the operational framework of privately funded operation home care businesses, which often cater to a defined demographic within a particular locale. Provision of services in accordance with ‘the Basic Norms for Home-Based Older adults Services’ (GB/T 43153–2023). It should be noted that if an organization provides home care services for the older adults in the form of a single service, a lot of problems may arise in the course of implementation. From the customer’s perspective, when multiple services are required simultaneously, they must sequentially order each individual service. Additionally, for customers with consistent and regular care requirements, repeated bookings of the same services are necessary. If they neglect to place an order, they forfeit access to the services, resulting in significant inconvenience and diminishing their overall experience. From the enterprise perspective, consistent long-term orders will substantially reduce the variability and unpredictability of orders, facilitating more precise allocation of service resources, effectively lowering operational costs, and enhancing the enterprise’s capacity to address spontaneous orders, thereby improving customer satisfaction. Consequently, for service programs characterized by elevated demand and increased frequency, it is essential to concurrently offer a composite service product comprising a range of individual services, specifically the older adult care package. To adequately meet the varied needs of senior citizens, different service packages might be concurrently launched, termed the older adults care service product family. It is often necessary to adjust the older adults care service product family offerings based on prevailing demand. The creation of older adults care service product families tailored to the specific regional context is a research area warranting significant attention.

It is important to recognize that the design of older adults care product families diverges from conventional product family design. Conventional product family design is an economical approach to assessing market demand, categorizing product components into modular types to create product family module libraries, configuring various series of segmented products from a shared platform in a market-driven manner, and producing a final product family to meet diverse customer segments (5). Traditional product family design contrasts with older adults care product family design in the following aspects. Primarily, the traditional approach is oriented toward the manufacturing sector, where products possess predetermined components and modules. Consequently, the final product family is generated solely through the combination of these fixed components and modules, which fails to address the needs of individual customers. The older adults care service project can be offered as an individual service addressing individual needs, while the older adults care service product family design caters to the requirements of diverse client segments. Secondly, the prior product family design just needs to account for the influence of external competition on the demand for the final product during product configuration; individual components do not possess market share and are not required to consider the competition among internal components. But older adults care services are presently experiencing an oversupply, with a restricted number of organizations offering such services in a specific area, and individual services can be delivered independently, necessitating consideration of the competitive influence of these individual services on the demand for service packages during the product family design for older adults care services. Third, prior product family design commenced with the identification of consumer perceptions and rival performance, quantitatively integrating these factors into product configuration design to link the demand and product configuration design stages for determining the product configuration aspect (6, 7). In the context of aging, the risk of market entry is unpredictable due to the specificity of the target demographic, and customer needs and preferences can only be anticipated through market research, which is constrained by technology and the particularities of the customer group in directly gathering expectation data. Compelling companies to tailor services to meet the needs of customers in particular regions. This approach mitigates resource wastage and excessive costs, necessitating more intricate design decisions for the development of products aimed at older adult services. In summary, the conventional product family design methodology is not entirely applicable to the design of product families for older adults care services. This research suggests a novel method to product family design for older adults care services.

## 2 Related work

### 2.1 Designing services for the older adults

The increasing population of older adult folks and the evolution of the ailments they encounter have necessitated enhanced social assistance and extended care services. Contemporary society emphasizes personalized care for older adult individuals, considering their distinct health problems, financial circumstances, and familial structures (8–10). Home older adults care services offer personalized care to address the varied needs of older persons, making the design of such services essential. Zhou et al. (11) propose that service design focused on older adults care can optimize the utilization of social resources to deliver precise and high-quality home care services for seniors in their residences. Consequently, home medical care, rehabilitation services, day care, in-home assistance, meal provision, bathing support, and other older adults care services are progressively becoming accessible to seniors, making a practical comprehension of their actual needs essential for addressing their requirements (12). Wei et al. (13) establish service design metrics for the rehabilitation requirements of the long-term care system, addressing the needs of long-term care stakeholders while also taking into account the emotional experiences of direct stakeholders, thereby enhancing the quality of care. Hu et al. (14) propose the reconstruction of functional service modules for semi-disabled older adult individuals within the community, establishing a health food system tailored to their needs, optimizing community resources, addressing the diverse healthcare requirements of the older adults, and offering an innovative perspective on the integration of medical care and older adults care within familial support systems (15). Chen et al. (16) used a service design methodology for an older adults demographic with mild cognitive impairment to summarize the hierarchy of high-level needs and guide design practice by observationally mapping user journeys, summarizing the service design strategies for mild cognitive impairment, and exploring more possibilities for design interventions for mild cognitive impairment. Thapaliya et al. (17) examine access to additional care services and the consumption of hospital and ambulance services by those utilizing the aging in place package, concluding that services must be tailored for this demographic to ensure sufficient support. The establishment of the home care service system constitutes a social system initiative. The persistent increase in demand for social pensions, coupled with constrained social resources, significantly hampers the advancement of social pension initiatives. Therefore, directing social organizations to offer home care services can effectively mitigate the pressures of social pensions while addressing the actual needs of the older adults, thereby promoting a diversified supply of older adult services (18–21). The design of older adult services in the process of continuous optimization of the home-based older adults service system can integrate older adults service resources from the needs of the older adults and improve the quality of life of the older adults.

### 2.2 Product family design

Product Family Design (PFD) is a strategy for expanding product offerings to satisfy a varied marketplace (22). Many businesses utilize product families and platform-based product development to address a broad spectrum of client requirements (23, 24). Liu et al. (25) consider the relative importance of components in responding to customer needs and the interrelationships between components to achieve optimal product architecture. To maintain market competitiveness, companies expand their product lines by launching product families; however, diverse customer needs cannot be sufficiently met through mass marketing strategies, highlighting the importance of product family positioning based on customer purchasing behaviors. Zhang et al. (26) proposed a fuzzy clustering-based market segmentation method that helps to effectively and efficiently plan the right product. The scope of the product family design issue was broadened to encompass the determination of suitable market positioning for each product within the family. Kumar et al. (27) proposed a new market-driven product family design (MPFD) approach to study the impact of increased variety in product offerings in different market segments and to explore the cost savings associated with communal decision-making. Xu et al. (28) proposed an information integration modeling architecture for the full life cycle of product families. Geng et al. (29) employed Quality Function Deployment (QFD) to translate demand attributes into engineering characteristics, thereby offering sustainable functional solutions for product design aligned with customer needs. In response to varied client expectations, abbreviated product development cycles, and cost constraints compelling manufacturing firms to adopt mass customization, product family design emerges as an effective strategy (30). The subsequent rise in the number of companies vying to provide a wide array of tailored services alongside customized products to enhance revenues and customer satisfaction. The application of new concepts such as service families and service platforms to the service industry through the use of the product family design methodology, the increasing diversity of service offerings leading to complexity and difficulty in estimating service costs. Tay et al. (31) proposed a service family cost estimation method based on service modularization and the job costing approach. Traditional product family design is a single-objective optimization problem. Du et al. (32) present a complex type of leader-following-joint optimization problem involving multiple decision makers encompassing different levels of decision hierarchies, consisting of many conflicting objectives competing to reach an equilibrium solution. Wu et al. (33) proposed an evolutionary planning model that has a stronger response to product evolution and can maximize firm performance in effective time. In response to escalating demand for personalization, a growing number of service businesses must provide customized service offerings while improving customer satisfaction and service quality in a cost-effective manner. The open product design allows for the incorporation of personalization modules into the product structure, thus meeting the unique requirements and preferences of individual clients. Tan et al. (34) proposed an optimization method that integrates individual consumer preferences via a genetic algorithm for individualized module allocation. Zhou et al. (35) proposed a hierarchical joint optimization model for the design of personalized service product families based on service resource families considering crowdsourcing of service operations, which was solved using a nested genetic algorithm to obtain a scheme for personalized service products. Feldman et al. (36) examined a classification optimization problem aimed at selecting the sequential presentation of products to maximize anticipated revenues. This approach breaks with the conventional product portfolio framework by focusing on the sequence of product portfolios presented in a customer-centric manner. Zhang et al. (37) consider uncertainty in service utility and customer behavior to maximize expected customer satisfaction as well as sales profit. Therefore, in the design of the service product family, we should focus on considering the customer’s preference, the probability of the customer’s choice of the products provided by the enterprise, and the related costs into the architecture system to ensure that the designed product architecture can be loved by the customer at the same time to achieve the goal of the enterprise to obtain profits.

A review of the literature on older adults care services and product family design indicates a significant rise in demand for services catering to the aging population, with home older adults care services demonstrating efficacy in mitigating the challenges faced by the older adults. To accommodate consumer preferences while minimizing operational expenses, product family design effectively addresses the elevated costs faced by companies due to the diverse customer requirements and the extensive range of home care services available in the market. This paper addresses the scarcity of studies on product family design for older adults care services by proposing a research methodology that prioritizes customer preferences for individual services. It emphasizes demand as a driving force, integrating the likelihood of customer selection of service products offered by the enterprise and associated costs into the design architecture. The approach aims to create a subset of service packages that maximizes profit while fulfilling the needs of the customer groups, concurrently managing individual services to address specific requirements, thereby enhancing the quality of life for the older adults and alleviating their challenges.

## 3 Principles and methods

### 3.1 Problem description

**Figure 1:**
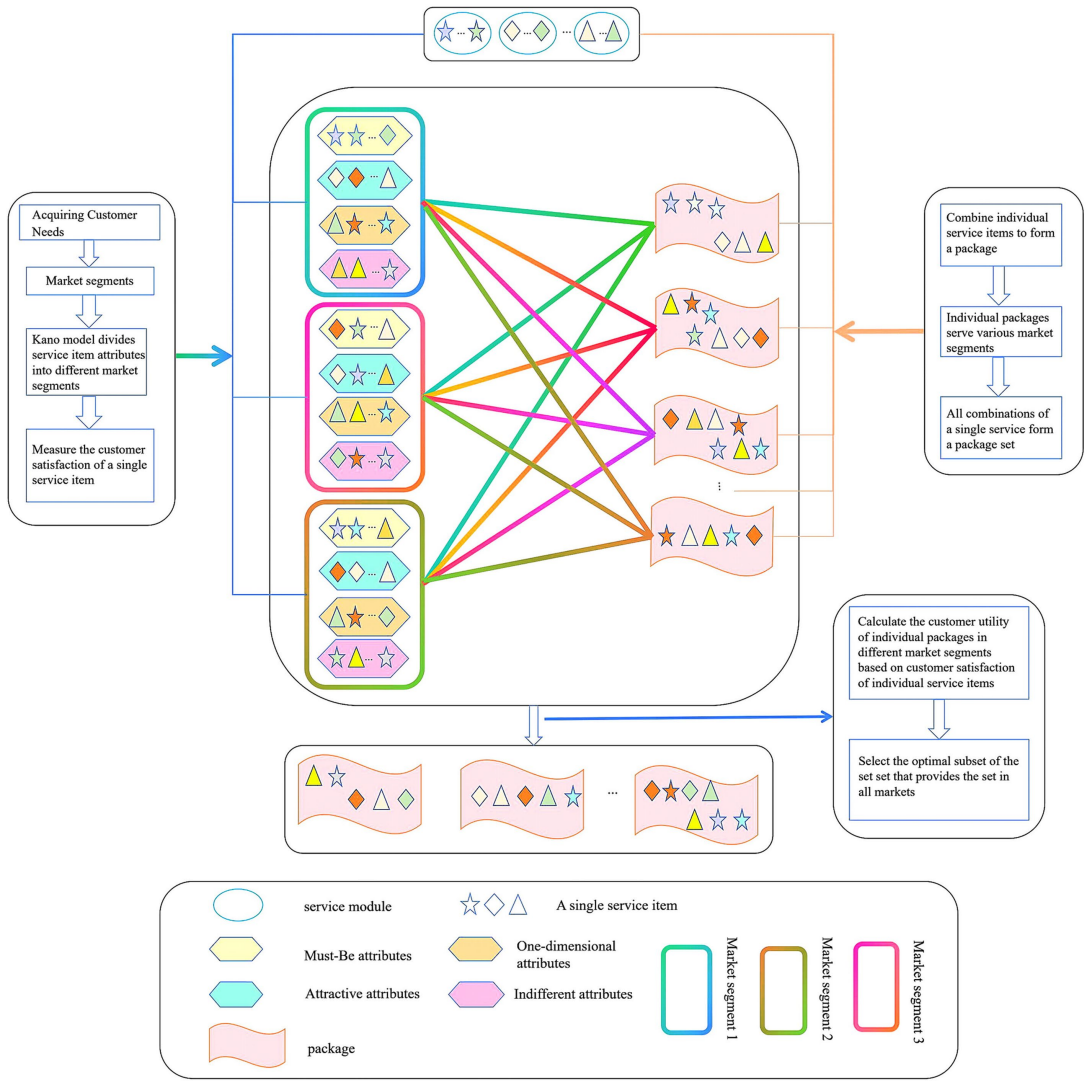
Design of a product family for older adult services.

This study addresses the optimization of the design of a range of older adults care service goods within a category of privately funded operation models. In this type of problem, the agency selects to offer in-home care services in a designated region with a known clientele size. After identifying the individual services in alignment with national standards, it amalgamates those with high demand and frequent delivery. Additionally, it evaluates the optimal array of older adults care service products from various options, constrained by limited resources. This article addresses the issue of the product family design program shown in Figure 1. This paper proposes a product family design for older adults care services that addresses the selection of an optimal package subset. The market offers a variety of service items, which can be categorized into distinct modules based on their functional characteristics, thereby organizing the service items systematically and enabling the segmentation of customer demand within the target market. According to the Kano model, the corresponding services under the service module can be categorized into attractive attributes $\left(A\right)$, one-dimensional attributes $\left(O\right)$, must-be attributes $\left(M\right)$ and irrelevant attributes $\left(I\right)$. Demand characteristics for individual services vary across distinct market segments. By combining service modules with corresponding service items, a package collection is created. Subsequently, packages are categorized based on price, allowing for the estimation of demand at each level within various market segments. The firm’s operational constraints are integrated by considering customer preferences, while essential configuration factors, including module type, customer demand, market competition for a singular service, selection probability, and variable versus fixed costs, are included to maintain the subset of packages that optimize the firm’s profitability during standard operations of a single service.

The symbolic description used to represent the sets and quantities involved in the design problem for this type of older adults care service product family is shown in Table 1.

**Table 1:** Symbolic description.

Mark	symbolic description
$\text{PM}=\left\{PM_{1},\ldots ,PM_{n}\right\}$	$PM_{i}$ denotes the ith module, i=1,2,…,n.
$SI_{i}=\left\{SI_{i1},\ldots ,SI_{im}\right\}$	$SI_{ij}$ denotes the jth service under the ith module, i=1,2,…,n, j=1,2…m.
${c_{ij}}^{V}$	Variable service cost of the jth service line under the ith module.
$c^{F}$	Fixed costs of package services provided by home care service companies.
$PF=\left\{PF_{1},\ldots ,PF_{g}\right\}$	$PF_{k}$ denotes the kth package, k=1,2,⋯,g.
$PF^{\prime }=\left\{PF_{1}^{\prime },\ldots ,PF_{g_{o}}^{\prime }\right\}$	$PF^{\prime }$ is a subset of PF.
$LE=\left\{LE_{1},\cdots ,LE_{h}\right\}$	$LE_{l}$ denotes the lth level, l=1,2,⋯,h.
$MS=\left\{MS_{1},\cdots ,MS_{f}\right\}$	$MS_{r}$ denotes the rth market segment r=1,2⋯f.
$q^{r}$	Number of sizes of the rth market segment.
$u_{0}^{r}$	Individual service utility added to the rth market segment outside the package.
$z_{ij}$	Monthly frequency of utilization of the jth service under the ith module.
$x_{kij}$	Binary integer = 1 or 0, If the jth service of the ith module of the kth package appears as 1, and if it does not appear as 0.
$y_{kij}^{M}$	Binary integer = 1 or 0, If the jth service of the ith module of the kth package is a must-be attribute, it is checked as 1 and unchecked as 0.
$o_{k}$	Binary integer = 1 or 0, if the k th package appears as 1 and if it does not appear as 0.

### 3.2 Gathering customer requirements

In reality, the service coverage area of an in-home older adults care business is established. Due to the variability in the physical conditions and service requirements of the older adults across different regions, it is imperative for each older adults care service organization to conduct a survey within its jurisdiction to assess the physical conditions and service needs of the older adult population, subsequently enabling the design of tailored older adults care service product families. A questionnaire survey was undertaken specifically for the older population in a designated area, employing a stratified random sampling method to guarantee that each eligible older adult individual had an equal opportunity to be picked for participation. The questionnaire was developed through interviews with pertinent service-oriented enterprises, examining the preliminary classification and statistical methodologies of service demand in the home care sector. The questionnaire was formulated following an extensive analysis of interview results, national standards for home care services, and variables such as the physical health, economic capacity, and age demographics of the older adults in the targeted operational region of the company. Due to the primary demographic of the questionnaire being the older adults, the execution of an online survey poses significant challenges. Therefore, a hybrid approach of both online and offline methods is employed. Additionally, considering the varying physical conditions of the older adults, those capable of completing the questionnaire independently may do so, while those facing difficulties may utilize either a question-and-answer format or have family members complete the questionnaire on their behalf.

**Figure 2:**
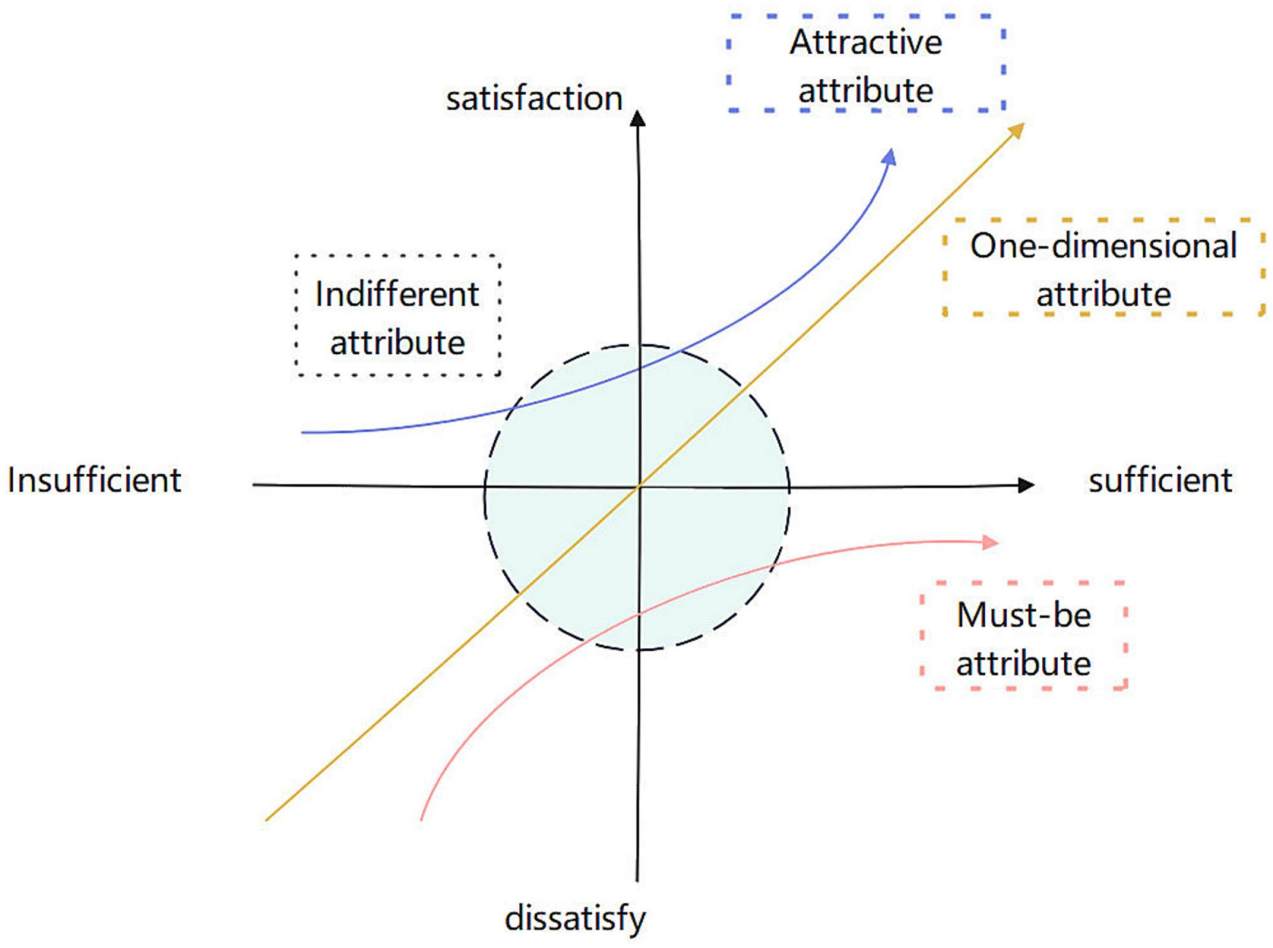
Kano model.

The data collected from the questionnaire was utilized to classify the characteristics of each service item using the Kano model. The must-be attributes of a service are the minimum requirements that must be met to provide basic life care. One-dimensional attributes are additional aspects of the service that add value and enhance the competitiveness of similar products after the must-be attributes have been satisfied. Attractive attributes are extra elements that aim to surprise and attract users. Irrelevant attributes are optional and do not significantly impact needs or preferences. Figure 2 displays the attributes of each category of demand in the Kano model. The horizontal coordinate is the level of sufficient, and the vertical coordinate is the level of satisfaction.

The questionnaire survey was conducted to assess the expectations of the senior population regarding different home services for the older adults. Let η be the size of the sample (38). The η calculation formula is shown in Equation 1.


(1) $$\eta =\frac{\mu_{\partial /2}^{2}\sigma^{2}}{\varphi^{2}}$$

$\mu_{\partial /2}$ is the standardized score. The value reflects the confidence level. φis the absolute error, the overall variance is $\sigma^{2}=\rho \left(1-\rho \right)$, ρ is the proportion of indicators. Upon determining the sample size, the questionnaire was disseminated to gather data. The collected data were then analyzed using SPSS 25 software to ensure that the questionnaire structure was reliable and valid. Descriptive statistical analysis was conducted to determine the proportion of relevant parameters.

### 3.3 Computation of utility functions

Based on the pre-processing of the questionnaires obtained from the acquisition of the needs of the older adults, the physical condition of the older adults in the region was analyzed, and the market was subdivided based on self-care into $MS=\left\{MS_{1},\cdots ,MS_{f}\right\}$. If the traditional Kano model is followed, the attributes of the service line are determined based on the attribute category that accounts for the most; many in-home senior care services have attractive attributes. This is not beneficial for evaluating the market and providing customers with tailored service programs that meet their actual needs. Therefore, it is important to introduce specific satisfaction and dissatisfaction indices for each service program in different market segments to assist in categorizing the types of demands. The satisfaction index of the jth service under the ith module under the rth market segment is represented by the variable $cs_{ij}^{r}$, whereas the discontent index is represented by the variable $cd_{ij}^{r}$. The $cs_{ij}^{r}$ and $cd_{ij}^{r}$ calculation formulas are shown in Equation 2 and Equation 3 (35).


(2) $$cs_{ij}^{r}=\frac{A_{ij}^{r}+O_{ij}^{r}}{A_{ij}^{r}+M_{ij}^{r}+I_{ij}^{r}+O_{ij}^{r}}$$

(3) $$cd_{ij}^{r}=\left(-1\right)\frac{M_{ij}^{r}+O_{ij}^{r}}{A_{ij}^{r}+M_{ij}^{r}+I_{ij}^{r}+O_{ij}^{r}}$$

$A_{ij}^{r},O_{ij}^{r},M_{ij}^{r},I_{ij}^{r}$ represent the number of services classifying the jth service under the ith module in the rth segment as attractive attributes, one-dimensional attributes, must-be attributes, and irrelevant attributes according to customer demand.

Due to the nascent nature of the home care service industry, the market system is imperfect. Additionally, traditional beliefs have led to a relatively low demand for home care services for the older adults. Hence, it is imperative to consider the magnitude of the satisfaction index and dissatisfaction index when assessing the level of customer satisfaction for each service item. This analysis is crucial for enhancing service quality and catering to consumer demands. The satisfaction level is determined by the distance from the origin to the point ($cs_{ij}$, $\left|cd_{ij}\right|$), where $cs_{ij}$ and $\left|cd_{ij}\right|$ are both between (0,1) and serve as the vertical and horizontal coordinates, respectively, (35), distance range between $\left(0,\sqrt{2}\right)$. In order to eliminate the effect of physical magnitude, the distance is mapped between (0,1) as the satisfaction value. Let ${s_{ij}}^{r}$ represent the customer satisfaction of the jth service within the ith module in the rth market segment, and let ${\hat{s}}_{ij}^{r}$ represent the value of the customer satisfaction mapping. The ${s_{ij}}^{r}$ and ${\hat{s}}_{ij}^{r}$ calculation formulas are shown in Equation 4 and Equation 5.


(4) $$s_{ij}^{r}=\sqrt{c{s_{ij}^{r}}^{2}+c{d_{ij}^{r}}^{2}}$$

(5) $${\hat{s}}_{ij}^{r}=\frac{s_{ij}^{r}}{\sqrt{2}}$$

A higher value of $s_{ij}^{r}$ indicates that the service has a stronger influence on user satisfaction and is of greater significance. The weight of each candidate module varies across different segments. The weight of a module is determined by standardizing the satisfaction rating of the service item associated with that module. Let $w_{i}^{r}$represent the weight of the ith module in the rth segment. The $w_{i}^{r}$ calculation formula is shown in Equation 6.


(6) $$w_{i}^{r}=\frac{{\hat{s}}_{\text{max}}^{r}-{\hat{s}}_{\text{min}}^{r}}{\sum_{i=1}^{n}\left({\hat{s}}_{\text{max}}^{r}-{\hat{s}}_{\text{min}}^{r}\right)}$$

${\hat{s}}_{\text{max}}^{r}=\max\left\{{\hat{s}}_{ij}^{r}|i=1,2,\cdots ,n,j=1,2,\cdots ,m\right\}$, ${\hat{s}}_{\text{min}}^{r}=\min\left\{{\hat{s}}_{ij}^{r}|i=1,2,\cdots ,n,j=1,2,\cdots ,m\right\}$, $\sum_{i=1}^{n}\left({\hat{s}}_{\text{max}}^{r}-{\hat{s}}_{\text{min}}^{r}\right)$ is the total of the differences between the highest and lowest levels of satisfaction in the related service items of all modules.

Enterprises will carefully evaluate the overall cost of providing services when operating service products. Decreasing service costs is beneficial in reducing the price of service products, thereby enhancing the competitiveness of enterprises. This increases the opportunities for enterprises to operate in emerging markets and mitigates market risks. Cost-based pricing is the simplest method of determining prices, where corporations set product prices based on the predicted profit margins associated with the cost of each service item (39). Let $p_{ij}$represent the price of the jth service within the ith module, taking into account the firm’s intended profit margin ε. The $p_{ij}$ calculation formula is shown in Equation 7.


(7) $$p_{ij}=\left(1+\varepsilon \right)c_{ij}^{V}$$

ε represents the anticipated profitability of the company, while $c_{ij}^{V}$ denotes the variable cost associated with each individual service item.

Adjusting the package price based on the economic capacity of the older adult population in the chosen area, examining the usage of individual services by customers through a questionnaire, and determining that the price of the package in various market segments also influences customer satisfaction. Consequently, customers have distinct target prices for packages, denoted as $p_{\text{aim}}$. Additionally, enterprises categorize packages as $LE=\left\{LE_{1},\cdots ,LE_{h}\right\}$ based on the price ceiling, which effectively guarantees the demand for these packages. The price of the package has an impact on the customer’s perceived utility. Each customer segment has a specific price p for the package. If the package price is higher than the target price, it will decrease customer satisfaction. Conversely, if the package price is lower than the target price, it will increase customer satisfaction. Therefore, the package target price can be used as a benchmark to assess the gains and losses (40). Let the benefit be represented by the $e_{k}$ and the damage be represented by the $b_{k}$. The $e_{k}$ and $b_{k}$ calculation formulas are shown in Equation 8 and Equation 9.


(8) $$e_{k}=\{\begin{array}{ll} 0, & p_{k}>p_{\text{aim}}^{l}\geq \sum_{i=1}^{n}\sum_{j=1}^{m}p_{ij}^{M}\\ 1, & p_{k}=p_{\text{aim}}^{l}\\ p_{\text{aim}}^{l}-p_{k}, & p_{k}<p_{\text{aim}}^{l}\leq \sum_{i=1}^{n}\sum_{j=1}^{m}p_{ij} \end{array}$$

(9) $$b_{k}=\{\begin{array}{ll} p_{\text{aim}}^{l}-p_{k}, & p_{k}>p_{\text{aim}}^{l}\geq \sum_{i=1}^{n}\sum_{j=1}^{m}p_{ij}^{M}\\ 0, & p_{k}=p_{\text{aim}}^{l}\\ 0, & p_{k}<p_{\text{aim}}^{l}\leq \sum_{i=1}^{n}\sum_{j=1}^{m}p_{ij} \end{array}$$

$\sum_{i=1}^{n}\sum_{j=1}^{m}p_{ij}^{M}$ is equal to the total of the prices of the must-be attribute items; $\sum_{i=1}^{n}\sum_{j=1}^{m}p_{ij}$ is equal to the total of the prices of all the service items; $p_{\text{aim}}^{l}$ is the price corresponding to the lth level. Based on prospect theory (41, 42), determine the prospect values of both gains and losses for each package. According to anticipated utility theory, preference homogeneity results in a consistent level of relative risk aversion. Let $v_{k}^{l\left(+\right)}$ represent the prospect value of gains and $v_{k}^{l\left(-\right)}$. The $v_{k}^{l\left(+\right)}$ and $v_{k}^{l\left(-\right)}$ calculation formulas are shown in Equation 10 and Equation 11.


(10) $$v_{k}^{l\left(+\right)}={\left(e_{k}\right)}^{\alpha }$$

(11) $$v_{k}^{l\left(-\right)}=-\lambda {\left(b_{k}\right)}^{\beta }$$

The variables α and β represent the level of concavity of the benefit and damage functions in the prospect function. These variables indicate the varying attitudes of decision makers toward benefits and harms. A higher value of variables α and implies that the decision maker is more inclined to taking risks, whereas the variable λ represents the level of aversion to losses of the decision maker. α=β=0.88, λ=2.25. These values are considered to be parameter values that can represent the approximate behavioral preferences of any decision maker (41–44). Let the prospect value of the package k be $v_{k}^{l}$, and let the combined prospect value of each package in different market segments be $a_{k}^{r}$. The $v_{k}^{l}$ and $a_{k}^{r}$ calculation formulas are shown in Equation 12 and Equation 13.


(12) $$v_{k}^{l}=v_{k}^{\left(+\right)l}+v_{k}^{\left(-\right)l}$$

(13) $$a_{k}^{r}=\sum_{l=1}^{h}{\hat{w}}_{l}^{r}v_{k}^{l}$$

${\hat{w}}_{l}^{r}$is the proportion of customers in the lth level under the rth segment to the total size of that market, and in order to eliminate the effect of the physical dimension, the combined prospect value should be between minimum damage and maximum gain. Mapping it between $\left(-1,1\right)$ and letting ${\hat{a}}_{k}^{r}$ be the mapped value of the composite outlook for the rth market segment as an influencing factor for adjusting the utility. Let the minimum damage be $a_{\text{min}}$. The maximum revenue for the rth market segment is $a_{\text{max}}^{r}$. The ${\hat{a}}_{k}^{r}$, $a_{\text{min}}$ and $a_{\text{max}}^{r}$calculation formulas are shown in Equation 14, Equation 15 and Equation 16.


(14) $${\hat{a}}_{k}^{r}=2\left(\frac{\left(a_{k}^{r}-a_{\text{min}}^{r}\right)}{a_{\text{max}}^{r}-a_{\text{min}}^{r}}\right)-1$$

(15) $$a_{\text{min}}=p_{\text{aim}}^{1}-\sum_{i=1}^{n}\sum_{j=1}^{m}p_{ij}$$

(16) $$a_{\text{max}}^{r}=p_{\text{aim}}^{h}-\sum_{i=1}^{n}\sum_{j=1}^{m}p_{ij}^{M^{r}}$$

$p_{\text{aim}}^{1}$ setting a minimum level for package. $\sum_{i=1}^{n}\sum_{j=1}^{m}p_{ij}$ represents the sum of prices for all service lines. $p_{\text{aim}}^{h}$ setting the highest level for the package. $\sum_{i=1}^{n}\sum_{j=1}^{m}p_{ij}^{M^{r}}$ is the sum of the prices of the service items in the rth market segment that are must-be attributes.

In practical scenarios, enterprises offering services must take into account customer preferences. Only when customer satisfaction reaches a certain threshold can the enterprise secure a more consistent demand for orders. This ensures that resources are not wasted due to supply–demand imbalances. Additionally, the enterprise must provide a range of essential services to meet customer expectations. Hence, in this work, the utility function is defined as the multiplication of the weight assigned to each candidate module included in the package of service items, together with the summation of the satisfaction level of each service item corresponding to the selected module and the extent of impact caused by the price. Customer satisfaction declines across different market segments if the jth service, which is a must-be attribute in the ith module of package k is not chosen. However, selecting this service does not provide any further benefit. Furthermore, the price of a package can significantly affect a customer’s satisfaction in different market segments. If the enterprise sets a price that is lower than the customer’s expectations, it will result in limited service options for the customers. Conversely, if the price offered by the company exceeds the client’s expected price, the customer will abandon the purchase due to budget constraints, leading to a loss of customer base. Therefore, the ideal strategy is to aim for a price closer to the goal price. Let $u_{k}^{r}$ represent the utility value of the kth package in the rth market segment. The $u_{k}^{r}$ calculation formula is shown in Equation 17.


(17) $$\begin{array}{l} u_{k}^{r}=\\ \sum_{i=1}^{n}\sum_{j=1}^{m}\left\{\left[w_{i}^{r}s_{ij}^{r}x_{kij}-o_{k}w_{i}^{r}s_{ij}^{r}\mu \left(1-{y_{kij}}^{M}\right)\right]/\sum_{i=1}^{n}\sum_{j=1}^{m}x_{ij}\right\}*\left(1+a_{k}^{r}\right) \end{array}$$

$w_{i}^{r}$denotes the weight of the ith module of the rth market, $s_{ij}^{r}$ denotes the customer satisfaction level of the jth service under the ith module of the r th market segment.

### 3.4 Determination of product requirements

The probabilistic choice rule is a more suitable method for estimating product demand, as it takes into account the size of the target market and the likelihood of customers choosing specific service offerings. This approach is more aligned with choice forecasting, making it probabilistic for customers to select packages. Within the collection of packages $\left\{PF_{1},\ldots ,PF_{g}\right\}$, each package is linked to a customer’s likelihood of selection, which is quantified in this study as the customer utility of the service item (34). Let $t_{k}^{r}$ represent the chance of selecting the kth package inside the rth market segment. The $t_{k}^{r}$ calculation formula is shown in Equation 18.


(18) $$t_{k}^{r}=\frac{\exp u_{k}^{r}}{\exp u_{0}^{r}+\sum_{k=1}^{g_{0}}\exp u_{k}^{r}}$$

$u_{k}^{r}$ represents the utility of the kth package for the rth market segment. $u_{0}^{r}$ is the utility of the single service chosen by the customer of the rth market segment. This utility should be a constant as the customer selects the single service based on their specific needs. In this study, the variable $u_{0}^{r}$ is determined by calculating the percentage of respondents who express their willingness to use an individual’s service in a survey. Within a specific market, the proportion of individuals willing to use each service at a specific price is calculated. These proportions are then added together and averaged to determine the utility value of a single service for customers in that market. $\sum_{k=1}^{g_{0}}\exp u_{k}$ is that the firm ultimately selects the most economically efficient subset of packages $\left\{PF_{1}^{\prime },\ldots ,PF_{g_{0}}^{\prime }\right\}$ from the complete set of packages.

The questionnaire data provides statistics that allow for determining the size of each market segment. Each segment will have a demand for the package. This paper estimates the demand for the package by multiplying the customer market size with the probability of choosing the package. Let $d_{k}$ be the market demand for the kth package. The $d_{k}$ calculation formula is shown in Equation 19.


(19) $$d_{k}=\sum_{r=1}^{f}q^{r}t_{k}^{r}$$

### 3.5 Profit function calculation

Profits in this study are defined as the difference between revenues and costs. Companies select a specific number of the most optimized subset of packages to offer. These packages combine various individual services and are available for customers to order every month within a particular budget. Each service has a different frequency of use within a month. Implementing monthly subscriptions facilitates cost efficiency, mitigates demand uncertainty, and enhances resource optimization. Companies offering packages to gain high demand will have a discount rate δ to reduce prices and attract more customers. Let the profit of the kth package be denoted as $pr_{k}$. The $pr_{k}$ calculation formula is shown in Equation 20.


(20) $$pr_{k}=d_{k}\left[\left(1-\delta \right)\sum_{i=1}^{n}\sum_{j=1}^{m}x_{kij}p_{ij}z_{ij}\right]-\sum_{i=1}^{n}\sum_{j=1}^{m}d_{k}{c_{ij}}^{V}x_{kij}z_{ij}-c_{k}^{F}$$

$p_{ij}$represents the price of the service item, $d_{k}$ represents the demand for the kth package, and $z_{ij}$ represents the frequency offered by the monthly package of service items.

### 3.6 Older adult service product family design optimization mode

Older adult service product family design is mathematically expressed as a mixed-integer programming model. The goal is to maximize profits by choosing the best combination of packages $\left\{PF_{1}^{\prime },\ldots ,PF_{g_{0}}^{\prime }\right\}$ to offer to customers. Profits are influenced by market demand, variable costs linked to care workers and materials, fixed expenses tied to equipment and staff involved in the provided packages, and the price of each service product. From the customer’s perspective, combining service products into a package is cheaper than purchasing each service product separately. The more variety of packages available, the higher customer satisfaction tends to be. However, as the number of package options increases, the demand for each package naturally decreases. When the demand cannot cover the cost of the package, the company’s profits will decline. From a firm standpoint, higher prices or lower costs associated with designing the senior care service product family can lead to increased profit margins. However, if the product is priced too high or specific features are compromised to cut costs, customer preference for the product and market demand will decrease. Comprehensive analysis: in order to maximize corporate profits, the enterprise must select a specific number of subsets of packages while ensuring market demand. The objective is to design an older adults service product family that takes into account the interaction between fixed costs, variable costs, product prices, and market acceptance, and to find an equilibrium point, establish a service product package structure model.


(21) $$\begin{array}{l} \max pr=\\ \sum_{k=1}^{g}o_{k}d_{k}\left[\left(1-\delta \right)\sum_{i=1}^{n}\sum_{j=1}^{m}x_{kij}p_{ij}z_{ij}\right]\\ -\sum_{k=1}^{g}\sum_{i=1}^{n}\sum_{j=1}^{m}o_{k}d_{k}{c_{ij}}^{V}x_{kij}z_{ij}-\sum_{k=1}^{g}o_{k}c_{k}^{F} \end{array}$$

(22) $$s.t.\sum_{k=1}^{g}o_{k}=g_{0},o_{k}\in \left\{0,1\right\},k=1,2,\cdots g$$

(23) $$\sum_{i=1}^{n}\sum_{j=1}^{m}|x_{kij}-x_{k^{\prime }ij}|>0,\forall k\neq k^{\prime }$$

Equation 21 refers packages are ordered on a monthly basis, the frequency of provision of individual service items in the monthly packages is determined to maximize the total profits of all packages. The variable cost is the direct cost assigned by the firm for offering individual services, and the total variable cost is determined based on the demand for the selected packages. The total fixed cost of a package offered by the enterprise remains constant regardless of market demand. It represents the constant expenses incurred by the enterprise in its regular operations, such as replacing old factory equipment with new ones and providing regular salary payments to management staff. By older adult service product family design, the enterprise ensures long-term stability and meets a certain level of demand to obtain profits. According to the changes in market size, the actual demand for packages will change. When the market size of the case increases, the actual demand for packages will increase, and the enterprise needs a larger site to provide services but also needs more caregivers; when the market size of the case is smaller, the situation is reversed. Based on the current status, enterprises can reduce wastage of resources such as personnel wages and rent by making efficient use of resources. This will help in reducing fixed costs and enable the enterprise to offer package deals at a lower price than individual services. Equation 22 indicates that the constraints applied to the optimization include limiting the number of package types in the product portfolio to $g_{0}$. Equation 23 restricts each package to be unique.

### 3.7 Model solving

The optimization model presented in this paper constitutes a single-objective optimization problem as the individual modules of the service items are selected randomly. Unlike other manufactured products, the composition of the package does not necessitate specific modules, and the service items associated with these modules are also chosen at random. There are no constraints on the number of modules included in the package or the corresponding service items. Consequently, when the quantity of service product modules and items is substantial, the problem’s size is demonstrated to be extensive through enumeration, categorizing it as an NP-hard optimization problem. Genetic Algorithm (GA) is very suitable for problems with large solution spaces and low efficiency of numerical algorithms.

**Figure 3:**
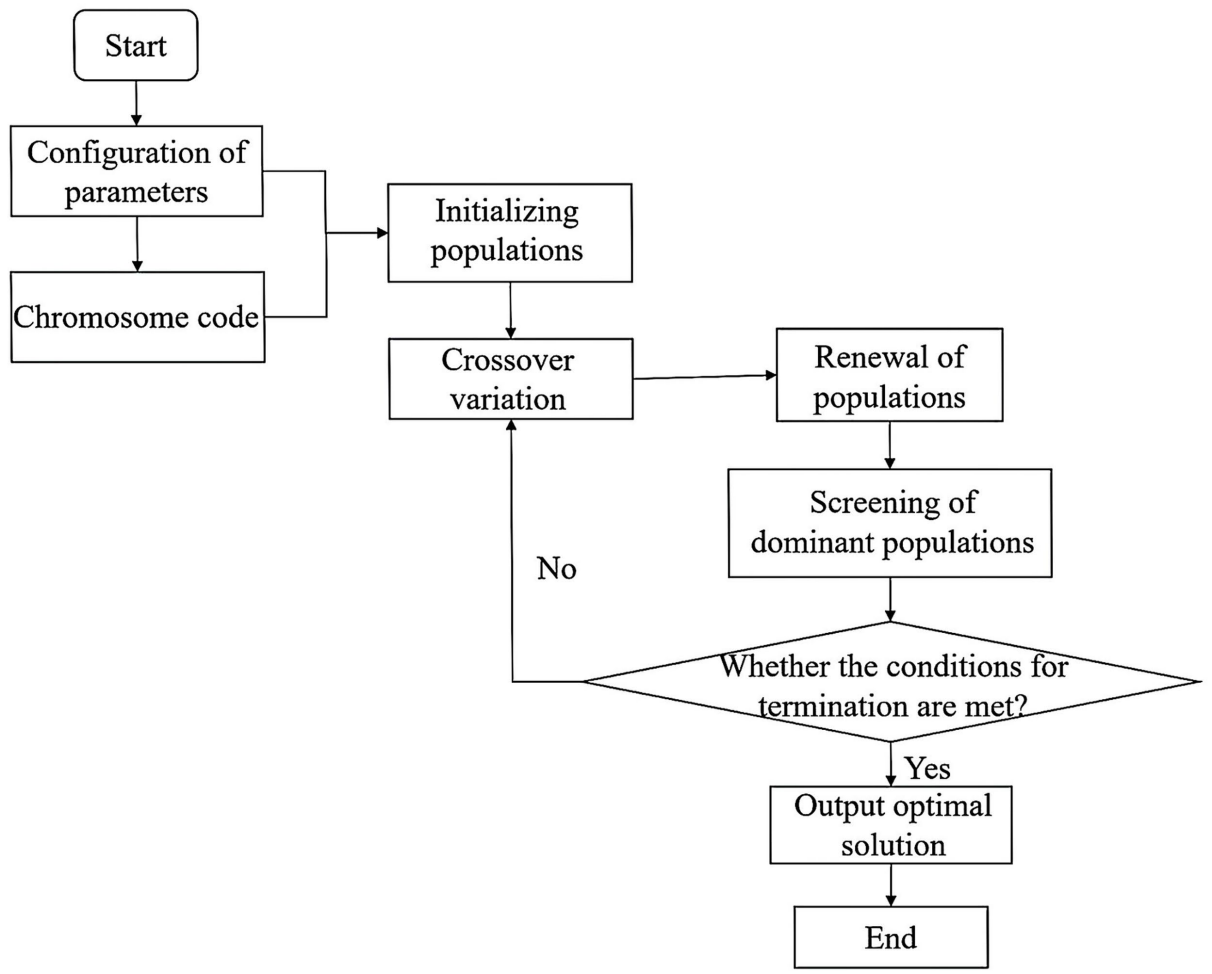
Genetic algorithm flow.

GA is widely used in many product family design problems (7, 34, 35, 37). This study presents a single-objective optimization genetic algorithm designed to solve the problem of older adult service product family design. The genetic algorithm flow is shown in Figure 3. The specific design steps of the genetic algorithm are as follows:

Step 1: Configuring the parameters. Specify the parameters for the product family design of the older adult service product, such as the number of modules and the matching list of service items for each module. Additionally, establish the population size, maximum number of iterations, crossover chance, and mutation probability.

Step 2: Chromosome encoding. Every chromosome is a binary sequence consisting of 0 and 1 that represents the composition of the package. The length of the chromosome is determined by the cumulative amount of service items across all modules. Each place on the chromosome can have a value of either 0 or 1, indicating whether the related service item is chosen or not.

Step 3: Initialize the population. Generate an initial population randomly, taking into account the decision variables of the optimization problem.

Step 4: Involves performing crossover and mutation operations. Tournament selection is employed to choose a fresh generation of people from the population that has undergone two-point crossover and bit-flip mutation processes. Specifically, in each round, the most successful individuals are chosen from a randomly chosen subset of the population.

Step 5: End the iteration. Firstly, ascertain whether the constraints are met. If they are, proceed to assess the fitness value of the individuals in the population. Secondly, verify if the satisfaction and gain have reached the maximum number of iterations. If they have, terminate the iterations and document the optimal solution and its corresponding value at that point.

## 4 Example analysis

### 4.1 Background and data

The case pertains to a home care service firm planning to establish a new home care service station on Huangcheng Street, Shenhe District, Shenyang City, encompassing all neighborhoods inside Huangcheng Street as its service area. China released ‘the Basic Norms for Home-Based Older adults Home Service’ (GB/T 43153–2023) on September 7, 2023. This standard delineates the overall requirements, service content, process and requirements, evaluation, and improvement for home-based older adult home service. It applies to organizations providing door-to-door service for older adults individuals in their homes, as well as their management. Furthermore, the standard categorizes the services into seven primary categories: life care services, basic care services, health management services, visiting and caring services, spiritual comfort services, commissioning services, and home living environment aging adaptation services. By modularization design and categorizing service items into modules based on the specific needs of the older adults, several benefits can be achieved. Firstly, it allows for a broader range of services, improved service quality, and the ability to meet diverse demands, enabling quick adaptation to market changes. Secondly, it facilitates a clear understanding of customer demands, making it easier to adjust service items through modularization and reducing operational costs for the enterprise. Each module is associated with specific service items, and Table 2 displays the older adults home care service items provided by the firm. To enhance customer satisfaction and decrease organizational operational expenses, it is planned to develop and introduce an older adult care service product line.

**Table 2:** Older adults home care service items.

Services modules	Module name	Service items	Service item name	Monthly package frequency
$PM_{i}$		$SI_{ij}$		
$PM_{1}$	life care services	$SI_{11}$	Home haircut	2 times per month
		$SI_{12}$	Home-based assistance with bathing	6 times per month
		$SI_{13}$	Home nail trimming	2 times per month
		$SI_{14}$	Delivery of meals (all-day meals)	20 times per month
		$SI_{15}$	Housekeeping services (laundry, bed making, cleaning)	10 times per month
		$SI_{16}$	Assistance with eating (all-day meals)	20 times per month
		$SI_{17}$	Assisting in the use of rehabilitation equipment	20 times per month
$PM_{2}$	basic care services	$SI_{21}$	Oral care	5 times per month
		$SI_{22}$	(Nursing assistance) Personal hygiene care	20 times per month
		$SI_{23}$	Excretory care (regular bowel movements)	20 times per month
		$SI_{24}$	Medication care	20 times per month
		$SI_{25}$	Rehabilitation Massage	15 times per month
		$SI_{26}$	Nighttime chaperone	20 times per month
$PM_{3}$	health management services	$SI_{31}$	Basic medical care (taking temperature, blood pressure, heart rate, blood sugar)	10 times per month
		$SI_{32}$	Health Consultation	5 times per month
		$SI_{33}$	Rehabilitation guidance to develop a program	2 times per month
$PM_{4}$	visiting and caring services	$SI_{41}$	Accompanying for chatting or chess	10 times per month
		$SI_{42}$	Accompanied walks or shopping	10 times per month
		$SI_{43}$	Accompanying for medical care	2 times per month
$PM_{5}$	spiritual comfort services	$SI_{51}$	Psychological guidance (adjusting the psychological state of the older adults through mental health education and psychological interventions)	5 times per month
		$SI_{52}$	Emotional counseling (talking and communicating with the older adults and listening patiently to them)	5 times per month
$PM_{6}$	commissioning services	$SI_{61}$	Purchase on behalf of others (purchase of daily necessities, booking of tickets, reservation of vehicles)	5 times per month
		$SI_{62}$	Representation (picking up and delivering letters, documents and goods, applying for legal aid, assistance services)	5 times per month
		$SI_{63}$	Payment on behalf of others (payment of daily expenses such as water, electricity, gas, communication fees, etc.)	5 times per month
$PM_{7}$	home living environment ageing adaptation services	$SI_{71}$	Bedroom adaptive retrofit planning	Free with monthly subscription package
		$SI_{72}$	Washroom adaptive retrofit planning	
		$SI_{73}$	Dining room adaptive retrofit planning	

To address the issue of older adults care service product family design, the following computational process and findings derived from the approach above for determining configuration possibilities are presented.

After an investigative visit, all communities in Huangcheng Street and conducting field interviews to ascertain that there are 5,771 older adults individuals aged 60 and above residing in the area. The study comprehensively considers the population of older adult residents, age distribution, physical condition, demand for older adults care services, and other relevant factors influencing the questionnaire’s formulation. The questionnaire was ultimately disseminated using stratified random selection to ascertain the senior group’s expectations for home care services. Due to the primary demographic of the questionnaire being the older adults, the implementation of an online format is more challenging; therefore, the preferred method is the physical distribution of the questionnaire. In light of the physical condition of the older adults, those capable of self-care may complete the questionnaire independently, whereas those requiring semi-self-care or unable to care for themselves may find it inconvenient to do so. In such cases, the questionnaire can be filled out through a question-and-answer format with the assistance of family members. Initially, based on the physical condition of the aged, the market is categorized into three segments: self-care, semi-self-care, and non-self-care. Subsequently, a confidence level of 90% is established. $\mu_{\partial /2}=1.645$. The absolute error φ is 5%, and to obtain a more conservative sample size, take ρ=0.5. The conservative sample size should be obtained by calculating 270 through Equation 1. Considering the questionnaire response validity rate of 90%, it was determined that the number of questionnaires to be collected after excluding the samples with missing research variables was 300. Furthermore, the data were examined based on the questionnaire outcomes utilizing SPSS 25.0. The overall questionnaire displayed a measured Alpha coefficient of 0.737. The forward questions exhibited an Alpha coefficient of 0.878, while the reverse questions displayed a coefficient of 0.825. These coefficients indicate a high level of confidence in the data. The subsequent KMO test and Bartlett test revealed that the overall questionnaire has a KMO coefficient of 0.753, indicating its suitability for factor analysis. The *p*-value for all cases was 0.000. The forward questions had a KMO coefficient of 0.844, also indicating suitability for factor analysis, with a p-value of 0.000. The reverse questions had a KMO coefficient of 0.778, indicating that they are better structured than the overall questionnaire, with a p-value of 0.000 in all cases. This suggests that the questionnaire possesses effective structure. The statistics of the basic information of the questionnaire are shown in Table 3, the level of the package’s segmentation by segments is shown in Table 4.

**Table 3:** The statistics of the basic information of the questionnaire.

Basic data	Categorize	Quantities	Ratios
Sex	male	174	58%
	women	126	42%
Age	60–69	94	31.3%
	70–79	136	45.3%
	80 or higher	70	23.4%
Residential situation	live alone	112	37.3%
	living with spouse	70	23.4%
	living with children	73	24.3%
	living with spouse and children	45	15%
Health condition	self-care	93	31%
	semi-self-care	164	54.7%
	non-self-care	43	14.3%
Utilization of services	unwilling	33	11%
	willing	267	89%

**Table 4:** The level of the package’s segmentation by segments.

Condition level	Self-care	Semi-self-care	Non-self-care	Total
Low-grade(¥260)	36(46.15%)	48(31.4%)	9(25%)	93(34.8%)
Mid-grade(¥350)	30(38.46%)	75(49%)	12(33.3%)	117(43.8%)
High-grade(¥500)	12(15.4%)	30(19.6%)	15(41.7%)	57(21.3%)
Total	78(29.2%)	153(57.3%)	36(13.5%)	267

**Figure 4:**
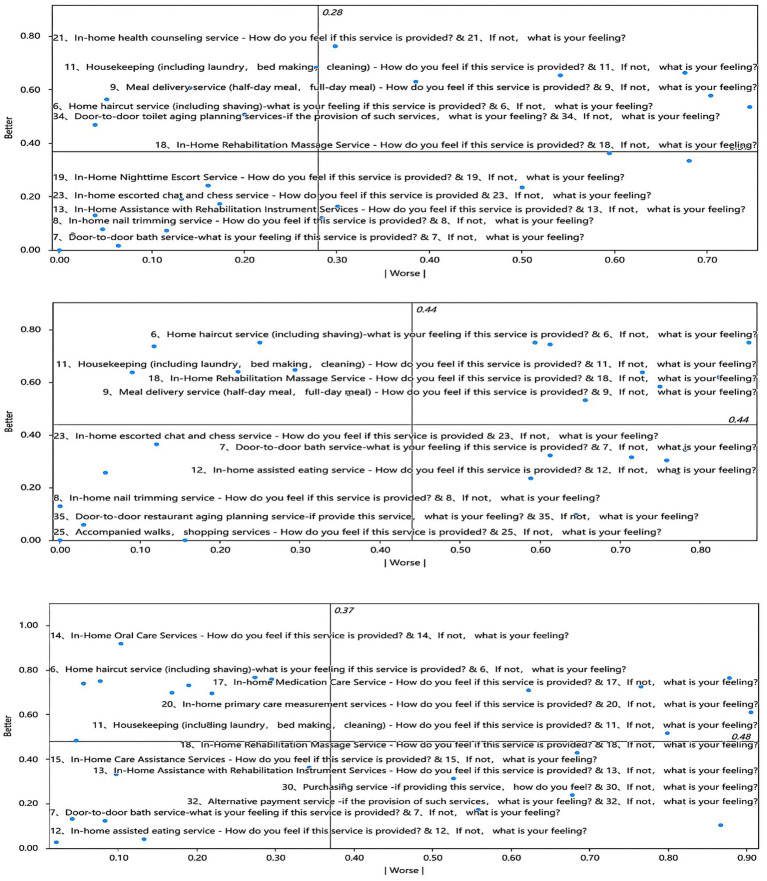
Classification of the Kano model for older adults individuals: self-care, semi-self-care and non-self-care.

Considering that the company’s provision of in-home elder care services is aimed at a certain group of customers who are open to using such services, 33 customers who expressed their unwillingness to utilize the services in the questionnaire were eliminated, resulting in a total of 267 valid surveys. According to the ratio of questionnaires received, there will be an estimated population of 5,771 individuals over the age of 60 residing on Royal City Street, with a potential customer base of around 5,136. The eligibility criteria and amount of subsidies for older adults home care services in the survey area are determined based on the guidelines provided in the notice of the ‘Shenyang Municipal Civil Affairs Bureau and Finance Bureau’ [Shenmin (2020) No. 18]. According to this notice, individuals who are over 100 years old receive a monthly subsidy of 800 yuan, while those between the ages of 90–99 receive 250 yuan per month. Additionally, older adult individuals between the ages of 80–89 who are classified as urban and rural low-income or low-income marginal recipients are eligible for subsidies through specific channels for low-income individuals. ‘Shenyang Circular of the Municipal Civil Affairs Bureau and the Municipal Finance Bureau on the Granting of Care Subsidies for the Older adults with Disabilities’ [Shenmin (2015) No. 80], ‘Shenyang Notice of the Municipal Bureau of Civil Affairs and the Municipal Bureau of Finance on Increasing the Standard of Care Subsidies for Older adults Persons with Disabilities and Semi-disabilities’ [Shenmin (2017) No. 222]. The latest standard for determining the disabled and semi-disabled care subsidy is a subsidy of 80 yuan per month for older adults people aged 60 or over in urban and rural low-income and marginal low-income households. According to the newest low-income standard, the minimum subsistence allowance for urban inhabitants in the Shenhe district is 1,065.65 yuan per month per household and 854.96 yuan per month per individual. Based on current local data, it is possible and viable to offer older adults home care services in Shenhe district, which is one of the busiest areas in Shenyang. This district is known for its commercial streets, making it the commercial hub of Shenyang. The region’s high purchasing power ensures that even low-income older adults individuals can afford these services. According to the Kano model, the older adults care services are classified based on their attributes. The satisfaction index $\left(cs_{ij}\right)$ and dissatisfaction index $\left(cd_{ij}\right)$ of each specific service item are calculated to determine the type of demand through Equations 2–5, with $cs_{ij}$ as the vertical coordinate and $\left|cd_{ij}\right|$ as the horizontal coordinate. The distance from the origin to the point ($\left|cd_{ij}\right|$, $cs_{ij}$) is used to measure the satisfaction level. Given the specific target market of the older adults, they will select different in-home services based on their physical conditions. To calculate satisfaction using the Kano model, the older adults are divided into three segments: self-care, semi-self-care and non-self-care. The resulting classification is shown in Figure 4. Service items in the first quadrant are classified as one-dimensional attributes, those in the second quadrant as attractive attributes, those in the third quadrant as irrelevant attributes, and those in the fourth quadrant as must-be attributes. The classification results and corresponding satisfaction values are presented in Table 5.

**Table 5:** Kano model classification.

Services modules	Service items	Market segment	$cs_{ij}$	$\left|cd_{ij}\right|$	Attributes	s
Life care services	Home haircut	self-care	50.67%	20.00%	I	0.5447
		semi-self-care	76.67%	27.33%	A	0.8140
		non-self-care	75.00%	59.37%	O	0.9565
	Home-based assistance with bathing	self-care	1.59%	6.35%	I	0.0655
		semi-self-care	12.41%	8.28%	I	0.1492
		non-self-care	32.26%	61.29%	M	0.6926
	Home nail trimming	self-care	7.81%	4.69%	I	0.0911
		semi-self-care	13.19%	4.17%	I	0.1383
		non-self-care	12.90%	0.00%	I	0.1290
	Delivery of meals	self-care	57.75%	70.42%	O	0.9107
		semi-self-care	76.35%	87.84%	O	1.1638
		non-self-care	53.13%	65.62%	O	0.8443
	Housekeeping services	self-care	65.28%	54.17%	O	0.8483
		semi-self-care	51.68%	79.87%	O	0.9513
		non-self-care	63.64%	72.73%	O	0.9664
	Assistance with eating	self-care	5.97%	1.49%	I	0.0615
		semi-self-care	4.20%	13.29%	I	0.1394
		non-self-care	23.53%	58.82%	M	0.6335
	Assisting in the use of rehabilitation equipment	self-care	12.16%	28.38%	I	0.3088
		semi-self-care	31.33%	52.67%	M	0.6128
		non-self-care	31.43%	71.43%	M	0.7804
Basic care services	Oral care	self-care	68.06%	27.78%	A	0.7351
		semi-self-care	91.72%	10.34%	A	0.9230
		non-self-care	75.00%	25.00%	A	0.7906
	Personal hygiene care	self-care	7.25%	11.59%	I	0.1367
		semi-self-care	36.36%	34.27%	M	0.4996
		non-self-care	9.68%	64.52%	M	0.6524
	Excretory care	self-care	0.00%	0.00%	I	0.0000
		semi-self-care	2.82%	2.11%	I	0.0352
		non-self-care	30.30%	75.76%	M	0.8159
	Medication care	self-care	62.86%	38.57%	O	0.7375
		semi-self-care	70.86%	62.25%	O	0.9432
		non-self-care	66.67%	36.36%	A	0.7594
	Rehabilitation Massage	self-care	36.23%	59.42%	M	0.6959
		semi-self-care	42.76%	68.42%	O	0.8068
		non-self-care	58.33%	75.00%	O	0.9501
	Nighttime chaperone	self-care	24.19%	16.13%	I	0.2907
		semi-self-care	28.28%	38.62%	M	0.4787
		non-self-care	25.71%	5.71%	I	0.2634
Health management services	Basic medical care	self-care	53.52%	74.65%	O	0.9185
		semi-self-care	60.81%	90.54%	O	1.0907
		non-self-care	61.76%	82.35%	O	1.0294
	Health Consultation	self-care	76.12%	29.85%	O	0.8176
		semi-self-care	75.84%	29.53%	A	0.8139
		non-self-care	74.19%	61.29%	O	0.9623
	Rehabilitation guidance to develop a program	self-care	60.56%	14.08%	A	0.6218
		semi-self-care	69.54%	21.85%	A	0.7289
		non-self-care	55.56%	36.11%	A	0.6626
Visiting and caring services	Accompanying for chatting or chess	self-care	17.33%	17.33%	I	0.2451
		semi-self-care	55.70%	21.48%	A	0.5970
		non-self-care	36.36%	12.12%	I	0.3833
	Accompanied walks or shopping	self-care	19.12%	13.24%	I	0.2326
		semi-self-care	33.33%	9.72%	I	0.3472
		non-self-care	0.00%	0.00%	I	0.0000
	Accompanying for medical care	self-care	66.20%	67.61%	O	0.9462
		semi-self-care	72.48%	76.51%	O	1.0539
		non-self-care	75.00%	86.11%	O	1.1419
Spiritual comfort services	Psychological guidance	self-care	53.42%	13.70%	A	0.5515
		semi-self-care	75.00%	7.64%	A	0.7539
		non-self-care	73.53%	11.76%	A	0.7446
	Emotional counseling	self-care	60.81%	14.86%	A	0.6260
		semi-self-care	69.80%	16.78%	A	0.7179
		non-self-care	63.89%	22.22%	A	0.6764
Commissioning services	Purchase on behalf of others	self-care	23.53%	50.00%	M	0.5526
		semi-self-care	23.97%	67.81%	M	0.7192
		non-self-care	34.38%	78.12%	M	0.8535
	Representation	self-care	33.33%	68.06%	O	0.7578
		semi-self-care	10.49%	86.71%	M	0.8734
		non-self-care	25.71%	77.14%	M	0.8131
	Payment on behalf of others	self-care	16.44%	30.14%	I	0.3433
		semi-self-care	17.24%	55.86%	M	0.5846
		non-self-care	0.00%	15.63%	I	0.1563
Home living environment ageing adaptation services	Bedroom adaptive retrofit planning	self-care	56.41%	5.13%	A	0.5664
		semi-self-care	73.79%	5.52%	A	0.7400
		non-self-care	63.64%	9.09%	A	0.6429
	Washroom adaptive retrofit planning	self-care	46.75%	3.90%	A	0.4691
		semi-self-care	72.97%	18.92%	A	0.7538
		non-self-care	64.71%	29.4%	A	0.7108
	Dining room adaptive retrofit planning	self-care	12.99%	3.90%	I	0.1356
		semi-self-care	48.34%	4.64%	A	0.4856
		non-self-care	5.88%	2.94%	I	0.0657

The cost vector for each modular service item provided by an in-home senior care provider is denoted as $c_{1}=\left(20.8,50,16.7,30,50,16.7,20.8\right)$, $c_{2}=\left(41.6,25,55,20.8,55,12.5\right)$, $c_{3}=\left(16.6,27.5,33.3\right)$, $c_{4}=\left(20.8,20.8,41.6\right)$, $c_{5}=\left(27.5,30\right)$, $c_{6}=\left(25,20,25\right)$, $c_{7}=\left(16.6,18.6,16.6\right)$. The fixed cost of providing a single package by a home care in-home service business is set at 100,000 yuan. Let the profit margin ε be 20% and the package discount δ be 10%. According to Equation 7, the individual price vector for each service item is calculated from the cost of the individual service item as $p_{1}=\left(24.96,60,20.04,36,49.92,20.04,24.96\right)$, $p_{2}=\left(49.92,30,66,24.96,66,15\right)$, $p_{3}=\left(19.92,33,39.96\right)$, $p_{4}=\left(24.96,24.96,49.92\right)$, $p_{5}=\left(33,36\right)$, $p_{6}=\left(30,24,30\right)$, $p_{7}=\left(19.92,22.32,19.92\right)$. The data collected from the questionnaire for the three market segments of the specific service items was used to calculate their satisfaction with the standardization process. According to the data obtained from the questionnaire for the three types of market segments of the specific service items through the Equation 6 to calculate their respective satisfaction with the standardization process to obtain the weights of the seven modules of each market segment are as follows: $w^{r=1}=\left(0.24,0.21,0.08,0.20,0.02,0.12,0.12\right)$, $w^{r=2}=\left(0.28,0.25,0.10,0.21,0.01,0.08,0.07\right)$, $w^{r=3}=\left(0.19,0.15,0.08,0.26,0.02,0.16,0.15\right)$. The firm categorizes the packages into low, medium, and high levels, and the target price vector for the level is $p_{\text{aim}}^{1}=260,p_{\text{aim}}^{2}=350,p_{\text{aim}}^{3}=500$, calculating the composite prospective value as an adjustment factor through Equations 8–16. Secondly, setting the corporate penalty coefficient μ=0.8, according to Equation 17 can calculate the utility value of each package in different segments. The utility value of a single service for each segment is estimated by the proportion of willingness to use a single service in the questionnaire, and the utility value of a single service in the three markets is 0.501, 0.491 and 0.499. By applying Equation 18, the probability of customers’ selecting each package is calculated for different market segments. The target market size is divided into 1,592, 2,809, and 735 individuals based on the market research ratio. The market is then consolidated using Equation 19 to determine the demand for each package across all markets. The profit value of each package is calculated using Equation 20. Finally, an optimization model is constructed using Equations 21–23 and solved.

### 4.2 Results and analysis

**Figure 5:**
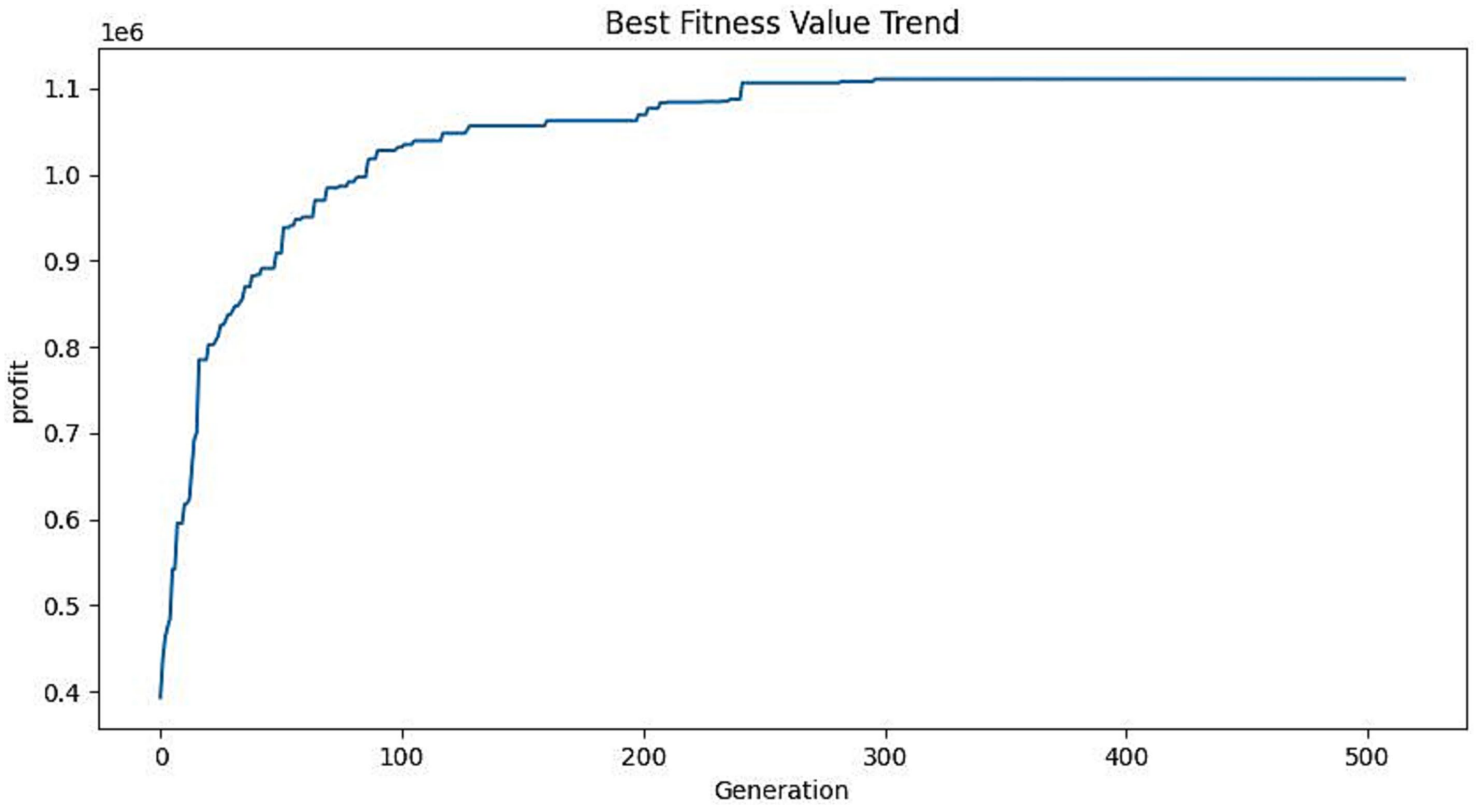
Iteration of target values.

**Figure 6:**
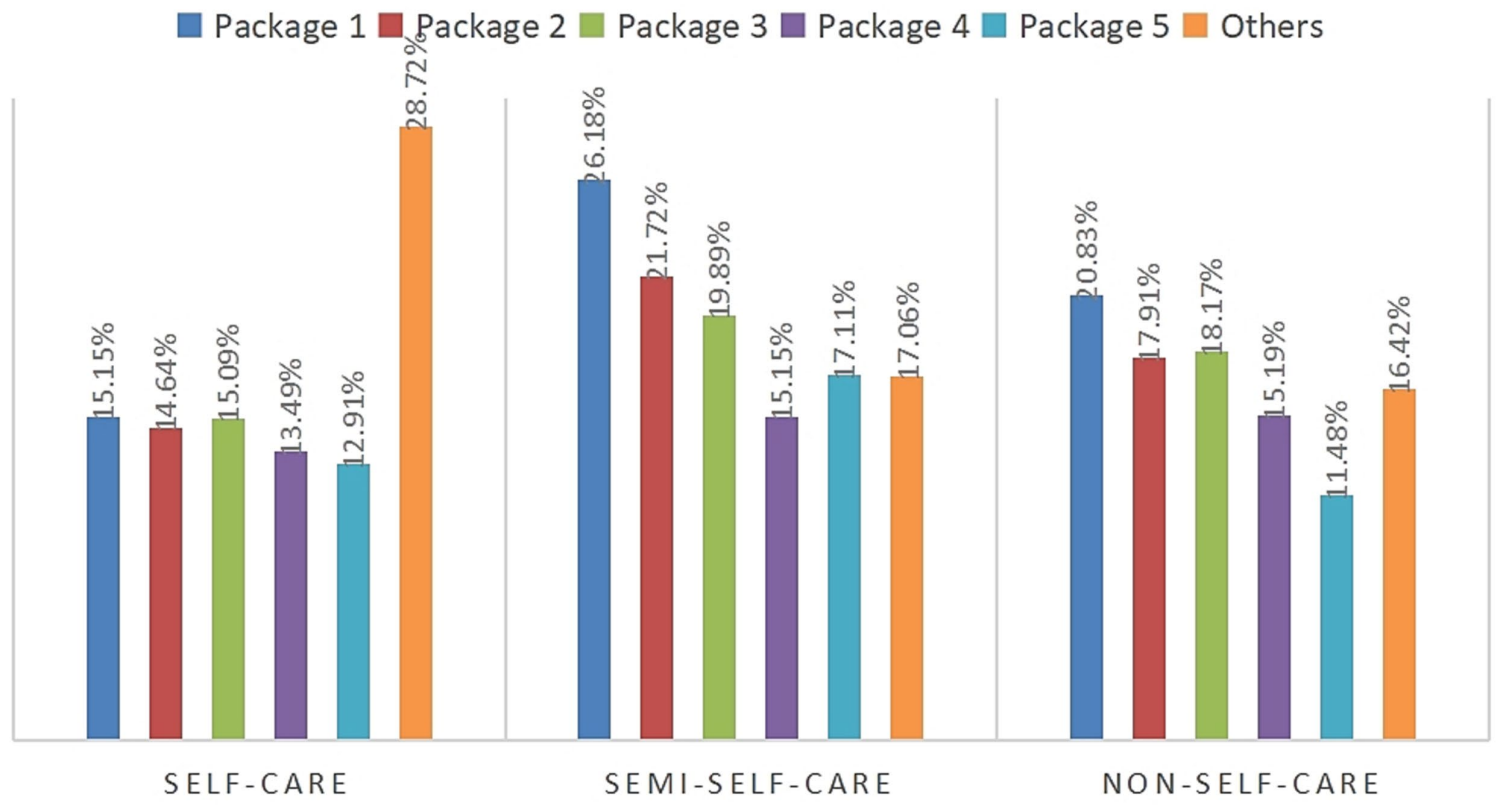
Probability of selection of packages in a market segment.

The evolutionary algorithm is used to optimize the selection of services included in packages by maximizing the profit generated by the firm. After taking into account the initial investment required for the company’s own activities, a total of five packages are chosen. The initialized population is set to 100, the crossover parameter interval is (0.4–0.99), and the initial crossover parameter is set to 0.5, the variation parameter interval is (0.001–0.1), and the initial variation parameter is set to 0.05. The crossover and variation probabilities are dynamically adjusted within the crossover and variation intervals by iteratively adjusting the crossover and variation probabilities in each generation. The algorithm is designed to balance between exploration and exploitation by adjusting probabilities. This prevents the algorithm from getting stuck in local optimums too early. As the iterations progress, the algorithm also adjusts crossover and mutation probabilities to maintain population diversity. This allows for the generation of new offspring and the calculation of their fitness values, which are then used in the next round of selection and evolutionary process. The algorithm runs for a total of 500 iterations. In this genetic algorithm, the selection of services is represented by a binary string. The final solution yields cross and variation parameters of 0.66 and 0.05. The design scheme for the older adults care services product family design program is presented in Table 6. The iteration of target values for profit maximization in the overall market for all packages is depicted in Figure 5. Additionally, the probability of selection for each package in the market segment is illustrated in Figure 6. The maximized profit for the subset of all optimal packages is 1,111,200 yuan. The profit vector obtained for each package is (¥334765.47, ¥262143.86, ¥214738.14, ¥167046.03, ¥132423.13). The price vector is (¥422.68, ¥422.76, ¥447.72, ¥397.8, ¥422.76). The probability of selecting packages in each market segment surpasses 70%, encompassing a significant portion of the market. This guarantees that the packages can secure a relatively consistent demand, thereby attracting customers and reducing costs. While obtaining a relatively stable order volume in the form of packages, the company operates individual services, increases the diversification of service types, reduces the risk of entering the market for home-based older adults care service enterprises, effectively understands the actual needs of the older adults, and provides the older adults with diversified services based on guaranteed profitability.

**Table 6:** Older adult care services product family design program.

Package category	Package items
Package 1	$\left\{SI_{11},SI_{12},SI_{14},SI_{21},SI_{22},SI_{23},SI_{24},SI_{25},SI_{26},SI_{31},SI_{41},SI_{42}\right\}$
Package 2	$\left\{SI_{11},SI_{12},SI_{14},SI_{21},SI_{22},SI_{23},SI_{24},SI_{25},SI_{26},SI_{41},SI_{42}\right\}$
Package 3	$\left\{SI_{11},SI_{12},SI_{14},SI_{21},SI_{22},SI_{23},SI_{24},SI_{25},SI_{26},SI_{42}\right\}$
Package 4	$\left\{SI_{11},SI_{14},SI_{21},SI_{22},SI_{23},SI_{24},SI_{25},SI_{26},SI_{42}\right\}$
Package 5	$\left\{SI_{11},SI_{14},SI_{21},SI_{22},SI_{23},SI_{24},SI_{25},SI_{26},SI_{43}\right\}$

## 5 Discussion

### 5.1 Influence of model parameters

**Figure 7:**
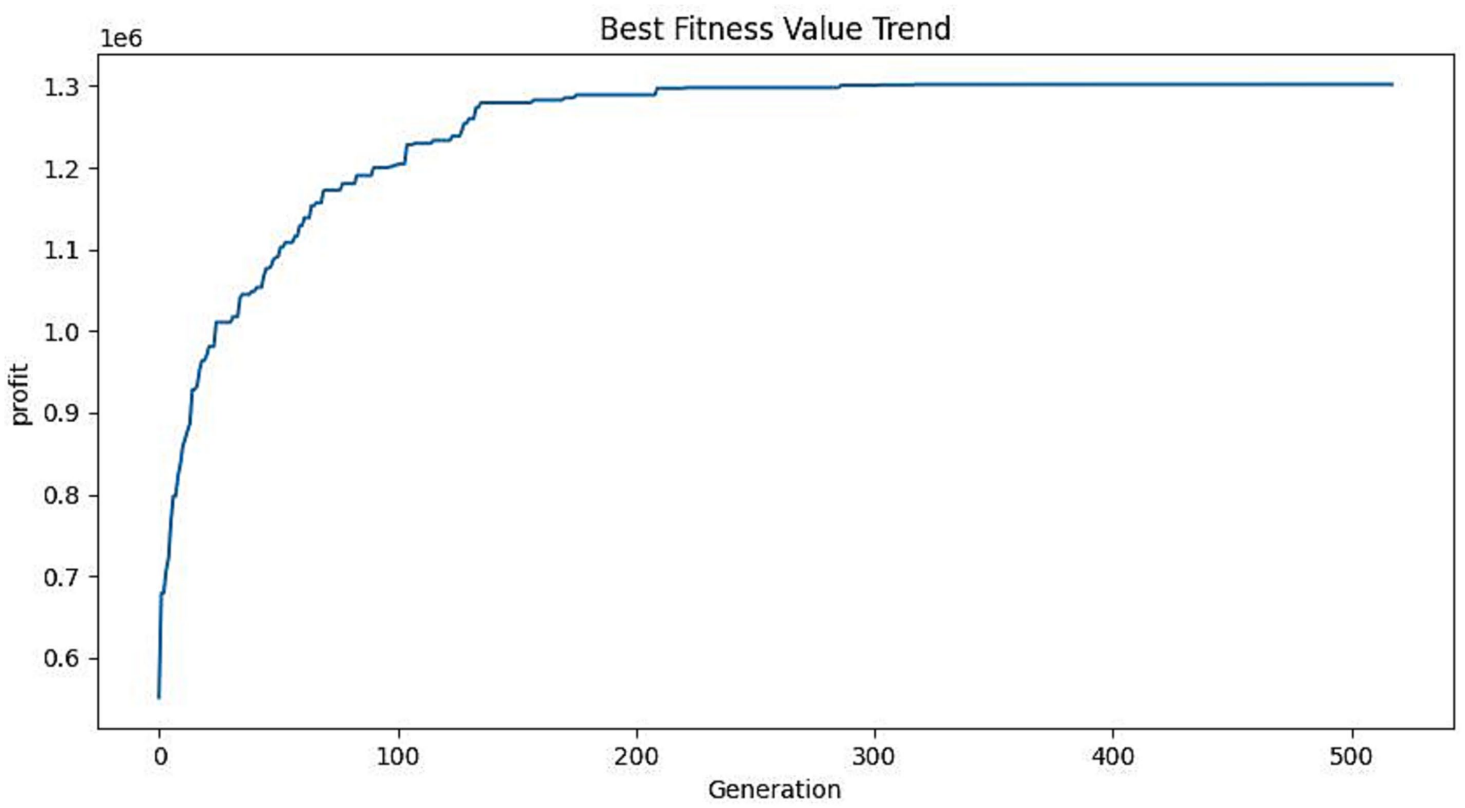
Iteration of target values.

This study employs a mixed-integer planning model to address the operational challenge faced by an in-home senior home care firm. The Kano model reveals that the demand for older adults care is primarily driven by the modules of life care and basic care services. By considering the unique preferences of the older adults in each market segment, packages can be designed to incorporate these services. This design process takes into account the operational constraints of the enterprise and addresses key configuration issues. The goal is to meet the actual care needs of the older adults while maximizing the enterprise’s profits. These results enable businesses to make real-time adjustments to the specific services offered. The chosen region has a direct impact on the operational limitations of the enterprise. In regions where the older adults have better financial circumstances and are more willing to pay for in-home older adults care services, the upper limit of the price for the package will increase. Assuming that each level increases in price by 100, the corresponding vector of target prices for the is $p_{\text{aim}}^{1}=360,p_{\text{aim}}^{2}=450,p_{\text{aim}}^{3}=600$, Figure 7 displays the progression of the objective value for maximizing profit in the whole market while keeping all other parameters constant. The maximum profit achieved is 1,319,000 yuan. The graded adjusted older adults care services product family design scheme is shown in Table 7.

**Table 7:** Graded adjusted older adult care services product family design program.

Package category	Package items
Package 1	$\left\{\begin{array}{l} SI_{11},SI_{12},SI_{14},SI_{16},SI_{21},SI_{22},SI_{23},\\ SI_{24},SI_{25},SI_{26},SI_{31},SI_{41},SI_{42} \end{array}\right\}$
Package 2	$\left\{SI_{11},SI_{12},SI_{14},SI_{21},SI_{22},SI_{23},SI_{24},SI_{25},SI_{26},SI_{31},SI_{41},SI_{42}\right\}$
Package 3	$\left\{SI_{11},SI_{12},SI_{14},SI_{21},SI_{22},SI_{23},SI_{24},SI_{25},SI_{26},SI_{41},SI_{42}\right\}$
Package 4	$\left\{SI_{11},SI_{12},SI_{14},SI_{21},SI_{22},SI_{23},SI_{24},SI_{25},SI_{26},SI_{42},SI_{43}\right\}$
Package 5	$\left\{SI_{11},SI_{12},SI_{14},SI_{21},SI_{22},SI_{23},SI_{24},SI_{25},SI_{26},SI_{43}\right\}$

Table 6 presents the optimal design program package for the senior care service product family under the $p_{\text{aim}}^{1}=260,p_{\text{aim}}^{2}=350,p_{\text{aim}}^{3}=500$ package price staging. In areas where the economic conditions for the older adults are favorable, there is a high acceptance of home care services, resulting in an increase in the upper limit of the package staging price. Table 7 indicates that, with all other parameters held constant, each increment of 100 in the package staging price affects the optimal design program packages for the senior care service product family under the target price of $p_{\text{aim}}^{1}=360,p_{\text{aim}}^{2}=450,p_{\text{aim}}^{3}=600$. Due to the increase in package level prices, there is heightened demand for services among the older adults, resulting in Table 7 reflecting one additional service per package compared to Table 6.

When comparing the results of two different grades, it is observed that the actual operating limitations of the enterprise are influenced by the chosen region. In various regions, the pricing changes based on different grades will impact the package composition due to the spending ability of the older adults. If the older adults have a higher economic status, opting for higher-priced grades will result in an increase in the number of service items included in the package, thereby boosting the enterprise’s profit. Conversely, if the older adults have low economic status, they will be less willing to pay for home care services, resulting in a decrease in the maximum price of the service package and the number of services included in the package. However, the government’s subsidy policy for economically disadvantaged older adult individuals will lead to variations in the service packages offered by different enterprises. Irrespective of the economic conditions and spending ability of the older adults, they have a preference for basic care services. Enterprises prioritize the supply of these services and subsequently include additional services based on the specific circumstances. The proportion of older adult individuals who require different levels of care (self-care, semi-self-care, unable to take care of themselves) varies across regions, affecting the packages offered. In conclusion, various regions must dynamically adapt the parameters of the design methodology outlined in this paper. This will ensure that the collection of packages selected in different situations enables the business to maximize profits effectively.

### 5.2 Impact of potential constraints

The proposed product family design for older adults care services in this study may encounter specific constraints that could influence the final composition of the implemented packages, as detailed below in relation to staffing and government regulation.

The staffing makeup may limit package composition, classified into two possibilities for targeted study. Firstly, if a home care service enterprise is not operational, with no services being provided and nursing staff unavailable, the service package may need to be adjusted due to staffing limitations in future operations, as the qualifications of nursing personnel differ based on the complexity of each service. In excretory and rehabilitative care, it is essential to carefully select nursing staff according to job descriptions to ensure their competencies fit with the position’s requirements. During the operational phase of the enterprise, there may be a deficiency of highly skilled nursing staff, according to the customer demand for the formation of packages, which will therefore temporarily not be able to provide. In the context where the home older adults care service firms discussed in this paper operate, the insufficient availability of high-level caregivers prevents the firm from offering multiple high-level services concurrently within a single package; thus, compatibility constraints are introduced while all other parameters remain constant. Incorporate a constraint $x_{k23}+x_{k25}+x_{k26}\leq 1,k=1,2,\cdots ,g$ into the product family optimization model for older adults care services established in Chapter 3. The package includes a maximum of one item from the three programs: defecation care, rehabilitation massage, and night escort. The product family design plan for older adults care services under this compatibility constraint is shown in Table 8, and the target value for profit maximization in the total market for all packages is 836,130 yuan. Consequently, it is essential to continually attract proficient nursing staff to enhance service provision while also considering the mobility of nursing personnel.

**Table 8:** Compatibility constrained older adults care services product family design program.

Package category	Package items
Package 1	$\left\{SI_{11},SI_{12},SI_{14},SI_{16},SI_{22},SI_{24},SI_{25},SI_{31},SI_{41},SI_{42},SI_{43}\right\}$
Package 2	$\left\{SI_{11},SI_{12},SI_{14},SI_{21},SI_{22},SI_{23},SI_{24},SI_{31},SI_{41},SI_{42},SI_{43}\right\}$
Package 3	$\left\{SI_{11},SI_{12},SI_{14},SI_{21},SI_{22},SI_{23},SI_{24},SI_{41},SI_{42},SI_{43}\right\}$
Package 4	$\left\{SI_{11},SI_{12},SI_{14},SI_{21},SI_{22},SI_{23},SI_{24},SI_{42},SI_{43}\right\}$
Package 5	$\left\{SI_{11},SI_{12},SI_{14},SI_{21},SI_{22},SI_{24},SI_{25},SI_{42},SI_{43}\right\}$

Secondly, if the home care service firm has initiated operations, each service is generally provided, and the nursing personnel are sufficiently equipped. Nevertheless, the package provided by the firm may be affected by the high expenses related to the actual delivery of specific services during its formulation. The main goal of design packages is to decrease cost for the firm while improving scheduling ease for caregivers. Moreover, in the implementation of specific packages, the firm seeks to provide uniform services. The necessary skill level of nursing staff differs among services, and the incorporation of specific services inside the package may necessitate the firm to allocate highly skilled nursing workers to provide lower-tier services, leading to elevated operational costs. The enterprise will consistently assess and modify staffing levels in accordance with the development strategy and fluctuations in demand for certain services. Moreover, the enterprise will routinely evaluate and modify staffing in alignment with its development strategy and fluctuations in demand for specific services, ensuring that staffing remains congruent with the development of the enterprise; this adjustment process may influence the composition of current packages. Consequently, in various scenarios, staffing will influence the package composition by the enterprise. To achieve sustainable development, it is essential to consider the impact of staffing on package composition during operations. The enterprise should integrate nursing staffing constraints into its actual development strategy to adapt the package composition flexibly.

Regulatory requirements will restrict package composition, which can be categorized into government regulation and internal enterprise regulation. Government regulation primarily involves the government assuming a leading role in regulatory responsibilities, with local governments tasked with comprehensive oversight of senior care service agencies within their jurisdictions. Their main responsibility includes enhancing supervision of practitioners and conducting regular inspections and evaluations to comply with government regulatory standards. Enterprises may need to modify their current individual services, leading to alterations in package composition. Internal supervision primarily involves the enterprise conducting rigorous quality control and assessing the nursing staff’s workflow, service demeanor, and competencies. In the execution of the package, evaluation outcomes and problem analysis inform regular rectification and enhancement of service quality, including the service items encompassed within the package. An excessive number of service items contained in the package may compromise service quality; therefore, pertinent supervisory constraints should be integrated into the rectification process and the package structure should be changed flexibly.

### 5.3 Extended application of the model

The proposed methodology for product family design in older adult services is also relevant to various other service sectors, including healthcare, home care, communication services, and other related fields. The following is an example to introduce the relevance of this paper’s method in the medical service industry in private medical examination institutions. In such institutions, individual examination items are often dispersed. When a patient requires multiple tests simultaneously, they must select each examination individually. This can cause much inconvenience to patients. In addition, setting different categories of standardized packages according to the age, gender, and occupational characteristics of the target population can facilitate the ordering of standardized packages for the same type of target patients and finally add additional individual medical checkups according to their needs. The stability of the standard package will effectively reduce the randomness and uncertainty of the order, enabling the firm to allocate resources more accurately and prevent resource wastage and customer attrition, thereby balancing supply and demand and effectively lowering operational costs. Concurrently, the price reductions of the standard package may, to some extent, increase demand, as patients will see the package cost as lower than that of a single checkup, thereby improving satisfaction. This approach will also facilitate the organization in elevating its overall service quality. In addition to the standard package, the organization offers individual physical examination programs, clearly delineating the specific costs of each examination alongside the total price of the package and transparently presenting this information to patients. Avoid concealed consumption or misleading patients, enabling them to select the most appropriate options based on their specific circumstances. Address the needs of the patient demographic while emphasizing the individualized requirements of personal checkups. Expand the demand for stable packages, augment the institution’s influence, and progressively train more qualified medical professionals to conduct and interpret reports, ensuring accuracy and reliability of results, thereby enhancing the medical quality and service standards of medical checkup institutions.

## 6 Conclusion

This paper presents a method for designing an optimal older adult care service product family that takes into account customer utility and enterprise operating profit. It achieves this by constructing and solving a model that maximizes enterprise profit, ultimately determining the design scheme for the older adults care service product family. In traditional product family design, products are categorized based on the production process, with fixed modules and optional personalized modules. However, in this paper, the service items in the modules of the service product family design are not limited to the process. Instead, they are focused on the functional characteristics. A greater degree of randomization exists between the modules and service items. Assessing the probability of package selection by examining internal competition among service items, forecasting potential client demands, and meticulously evaluating and standardizing customer criteria designed to fulfill client requirements in the market. Based on this analysis, we design a set of service packages that are relevant to these customer groups. Simultaneously, in the context of aging, random pension service projects are more dispersed, based on the diversity of home care services. Through the design of the older adults care service product family, single service combinations will be dispersed in order to ensure the demand at the same time to save the operating costs, endowed with the enterprise to increase the service diversification of the guarantee. By packages utilizing service resources and staffing, the aim is to prevent resource wastage and costly configurations. The package ensures a stable order by effectively coordinating the balance between service supply and demand. This prevents the loss of customers due to excessive demand caused by resource shortages, as well as mitigates the risk of high operating costs resulting from excess resources due to insufficient demand. Achieving a balance in service supply and demand facilitates efficient resource scheduling and enhances service quality for the enterprise. Ensuring a balance between service supply and demand enables more efficient resource scheduling, boosts service quality, guarantees timely service delivery, and increases customer satisfaction. This paper proposes a new method to optimize the product family design of older adults home care services. Identify the optimal combination of packages tailored for the designated area by home care service providers to fulfill customer group requirements, thereby securing consistent orders to mitigate market risks and achieve the goal of maximizing corporate profits. These packages should be structured to reduce operational costs while ensuring the uninterrupted delivery of individual services, addressing both group needs and individual demands in real-time. Better cope with the rapid development of aging and the growing care needs of the older adults, effectively alleviate the pressure on the older adults, and improve the efficiency of social pension.

With the design of a family of older adults service products that consolidates various service offerings into packages and provides individual services, enterprises can effectively reduce operational costs, enhance service quality, and attract a larger older adult clientele, thereby establishing a more stable customer base. This approach allows for the strategic hiring of personnel and resource utilization based on the packaged services. Customers can access the service at a reduced rate compared to the standard price, and acquiring the service as a package streamlines the selection process, allowing the enterprise to allocate resources effectively, thus expediting service delivery and ensuring punctuality. Offering an innovative approach to addressing service design challenges in practice. It also serves as a theoretical reference for the operational strategies of home care service-oriented enterprises, enabling them to effectively respond to the swift progression of aging and the escalating care demands of the older adults. Furthermore, this design methodology is applicable to related domains, such as housekeeping service.

This article has three primary limitations. The first point is that differing physical conditions necessitate distinct service quality standards, and the costs of service programs differ across various demographics. This paper employs a simplistic cost-based pricing approach, which inevitably results in approximations and inaccuracies that may cause the final profit estimates to deviate from their actual values. In the future, more precise pricing methodologies could be integrated to account for the multifaceted impact of services on costs, thereby enhancing alignment with actual values in design. The second point is that this paper exclusively examines the internal competition of a singular service product within the operational framework of an enterprise. However, actual enterprise operations may necessitate consideration of external competitive service products due to regional disparities. Adjustments should be made in accordance with the prevailing environment to enhance the accuracy of demand estimations and mitigate operational risks. The third point is that the scenarios incorporated in the model’s design in this paper are more idealized and do not thoroughly examine the impacts of policy. The specific policies of the Chinese government’s healthcare decision-making bodies in a particular region must be taken into account during implementation to enhance the model’s detail and comprehensiveness.

## Data Availability

The original contributions presented in the study are included in the article/supplementary material, further inquiries can be directed to the corresponding author.
